# Use of Two Self-referral Reminders and a Theory-Based Leaflet to Increase the Uptake of Flexible Sigmoidoscopy in the English Bowel Scope Screening Program: Results From a Randomized Controlled Trial in London

**DOI:** 10.1093/abm/kax068

**Published:** 2018-01-15

**Authors:** Robert S Kerrison, Lesley M McGregor, Nicholas Counsell, Sarah Marshall, Andrew Prentice, John Isitt, Colin J Rees, Christian von Wagner

**Affiliations:** 1 Research Department of Behavioural Science and Health, University College London, London, UK; 2 Cancer Research UK & UCL Cancer Trials Centre, University College London, London, UK; 3 St Mark’s Bowel Cancer Screening Centre, St Mark’s Hospital, Middlesex, UK; 4 Partners in Creation, Top Studio, London, UK; 5 South Tyneside NHS Foundation Trust, South Tyneside School of Medicine, Pharmacy and Health, Durham University, Durham, UK

**Keywords:** Colorectal cancer, Screening, Uptake, Flexible sigmoidoscopy, Behavioral science

## Abstract

**Background:**

We previously initiated a randomized controlled trial to test the effectiveness of two self-referral reminders and a theory-based leaflet (sent 12 and 24 months after the initial invitation) to increase participation within the English Bowel Scope Screening program.

**Purpose:**

This study reports the results following the second reminder.

**Methods:**

Men and women included in the initial sample (*n* = 1,383) were re-assessed for eligibility 24 months after their invitation (12 months after the first reminder) and excluded if they had attended screening, moved away, or died. Eligible adults received the same treatment they were allocated 12 months previous, that is, no reminder (“control”), or a self-referral reminder with either the standard information booklet (“Reminder and Standard Information Booklet”) or theory-based leaflet designed using the Behavior Change Wheel (“Reminder and Theory-Based Leaflet”). The primary outcome was the proportion screened within each group 12 weeks after the second reminder.

**Results:**

In total, 1,218 (88.1%) individuals were eligible. Additional uptake following the second reminder was 0.4% (2/460), 4.8% (19/399), and 7.9% (29/366) in the control, Reminder and Standard Information Booklet, and Reminder and Theory-Based Leaflet groups, respectively. When combined with the first reminder, the overall uptake for each group was 0.7% (3/461), 14.5% (67/461), and 21.5% (99/461). Overall uptake was significantly higher in the Reminder and Standard Information Booklet and Reminder and Theory-Based Leaflet groups than in the control (odds ratio [OR] = 26.1, 95% confidence interval [CI] = 8.1–84.0, *p* < .001 and OR = 46.9, 95% CI = 14.7–149.9, *p* < .001, respectively), and significantly higher in the Reminder and Theory-Based Leaflet group than in the Reminder and Standard Information Booklet group (OR = 1.8, 95% CI = 1.3–2.6, *p* < .001).

**Conclusion:**

A second reminder increased uptake among former nonparticipants. The added value of the theory-based leaflet highlights a potential benefit to reviewing the current information booklet.

**Trials Registry Number:**

ISRCTN44293755.

## Teaser

Reminder letters, which prompt ‘no-shows’ to self-refer for bowel cancer screening, increase uptake and thereby the total number of pre-cancerous lesions detected.

## Introduction

Colorectal cancer is a leading cause of morbidity and mortality throughout the world [1]. Several large randomized controlled trials have shown that a single flexible sigmoidoscopy screen between the ages of 55 and 64 can significantly reduce the incidence and mortality of the disease among people who complete the test [2]. As a result, several countries have begun piloting flexible sigmoidoscopy-based screening programs for the prevention of colorectal cancer [3], with England currently rolling out a national program (referred to as the Bowel Scope Screening program) set to reach full population coverage in 2018.

One of the key determinants of successful screening programs is the ability to achieve high population uptake. In England, all screening and treatment is offered automatically and free of charge through the National Health Service. However, despite being offered automatically and for free, the uptake of bowel scope screening is both low and socioeconomically graded [4]. One recent study found that only 43% of men and women invited for bowel scope screening during the initial implementation of the program attended an appointment, and that uptake was lowest among individuals living in the most deprived areas (uptake ranged from 32% in the most deprived areas to 52% in the least deprived) [4]. This is not a problem exclusive to the UK [5]. In the USA, for example, nearly half (48%) of eligible adults are not up to date with screening recommendations, despite available guidelines and evidence demonstrating their effectiveness [6].

As with other screening programs, the National Health Service bowel scope screening program incorporates specific strategies to maximize uptake (e.g., prenotification letters, reminder letters, timed appointments) [7–9]. Invitees receive a prenotification letter shortly after their 55th birthday. They then receive an invitation with a timed appointment 2 weeks thereafter. Anyone who does not respond to their invitation within 2 weeks is sent a reminder. If there is no response within an additional 2 weeks, the appointment is cancelled, and the individual is notified via direct mail. Anyone who confirms an appointment, but does not attend, is similarly notified. In both cases, the recipient is informed that they can self-refer for bowel scope screening up until age of 60, when they are eligible for a fecal occult blood test once every 2 years up until the age of 74.

Previous research exploring nonparticipation and decision making in the English Bowel Scope Screening program has identified a number of barriers to uptake, including “a perceived or actual lack of need to have the test”, “an inability to attend the appointment offered”, and “a lack of understanding about the harms and benefits of screening” [10]. One of the subsequent suggestions to improve uptake has been to send nonparticipants an additional reminder at a later date [10], and already there is some evidence to suggest that this may be effective [11].

We ourselves have previously examined the feasibility of sending bowel scope screening nonparticipants a reminder letter and leaflet 12 months after their initial invitation [11]. More specifically, we have previously investigated the feasibility of sending nonparticipants a theory-based leaflet (designed according to principles put forth by the Behavior Change Wheel) [12] and reminder letter (hereafter referred to as a “self-referral reminder”) that gave instructions for how to self-refer and included options for the day and time of the appointment and the gender of the practitioner performing the test [11]. On the basis that: (i) the reminder letter and leaflet could be implemented and (ii) would be more effective if sent a second time (i.e., 24 months after the initial invitation) [13–15], we performed a formal randomized controlled trial to test their effectiveness against usual care (i.e., no reminder).

Results from the first stage of the randomized controlled trial (i.e., the first reminder) demonstrated that sending nonparticipants a single self-referral reminder, 12 months after their initial invitation, significantly increased participation against usual care, and that reminders were more effective when sent with the theory-based leaflet, as opposed to the standard information booklet used by the bowel scope screening program [16]. Results from the second stage of the randomized controlled trial have not previously been examined.

This study reports the “additional” and “overall” uptake of bowel scope screening following the second reminder. Our specific aims were to (i) examine whether a second self-referral reminder increased the uptake of screening among former nonparticipants; (ii) assess the cumulative effect of the two self-referral reminders combined; and (iii) test whether the effect of the theory-based leaflet on participation was sustained after the delivery of a second reminder.

## Methods

### Study Population, Design, and Trial Setting

We performed a single-blinded, randomized, controlled trial with three parallel arms in the London boroughs of Brent and Harrow. One thousand three hundred and eighty-three men and women randomly selected from a weekly variable total of nonparticipants were randomized (using simple pseudo-random allocation methods) to receive either (1:1:1) no reminder (control, *n* = 461), a 12-month self-referral reminder and standard information booklet (Reminder and Standard Information Booklet, *n* = 461), or a 12-month self-referral reminder and theory-based leaflet designed using the Behavior Change Wheel (Reminder and Theory-Based Leaflet, *n* = 461). Anyone who did not attend an appointment within 12 weeks of being sent the 12-month reminder (or no reminder in the case of the control) was re-assessed for eligibility 24 months after their initial invitation (i.e., 12 months after the first reminder). Individuals who had (i) taken part in screening, (ii) registered with a general practice outside of the London boroughs of Brent and Harrow, or (iii) died were excluded. The remaining population were considered “eligible” and assigned to receive the same treatment they received 12 months previous.

Because individuals were assigned to receive no reminder or a self-referral reminder with one of two leaflets, it was not possible to blind them to the treatment they received. In terms of the study setting, the London boroughs of Brent and Harrow have below-average uptake and contain some of the most ethnically diverse and socioeconomically deprived areas in England [17].

### Procedures

Eligibility was re-assessed using routine data stored on the National Health Service Bowel Cancer Screening System: an electronic system that provides up-to-date uptake data for individuals enrolled in the national screening program [18]. Individuals in both reminder groups were able to book an appointment by returning an “appointment-request-slip” to St Mark’s Bowel Cancer Screening Centre (the screening center where appointments for people living in Brent and Harrow take place), thereby initiating a call from a member of the administrative team to arrange an appointment, or by calling the screening center directly on the Freephone number provided in the reminder letter. Anyone not responding to the “24-month” self-referral reminder within 4 weeks was sent a “follow-up” reminder, which also included an appointment-request slip, the allocated information leaflet, and a Freepost return envelope addressed to St Mark’s Bowel Cancer Screening Centre. Individuals were given an additional 8 weeks to respond before their attendance was assessed on the Bowel Cancer Screening System. Anyone referring for an appointment after this time was excluded from the study results, but was still offered an appointment. Individuals who referred for bowel scope screening were sent a pre-appointment text message and telephone call (where a mobile/home telephone number was available), as per routine practice.

### Intervention Development

The intervention strategy was informed by the Behavior Change Wheel [12], which was used (in conjunction with the Behavior Change Technique Taxonomy [19]) to identify the putative targets for change and the behavior change techniques likely to affect those targets. We began by defining the problem in behavioral terms (see online Supplementary material for the completed worksheets), before selecting and specifying the target behavior and identifying what needed to change (in COM-B terms) for the behavior to occur. We then identified the intervention functions and policy categories that would be most likely to bring about the desired change and reviewed the possible behavior change techniques and modes of delivery that could be used to deliver them.

After identifying the intervention strategy (Table 1), we developed the intervention content. We did this by the following methods: (i) reviewing the literature examining the perceived barriers and benefits of screening, (ii) interviewing previously screened adults, and (iii) contacting the local primary care cancer leads to obtain a local primary care endorsement. An overview of these activities and how they were used to develop the intervention content/deliver the behavior change techniques underpinning the intervention strategy is provided in Table 2.

**Table 1:** Summary of the intervention strategy arrived at through the behavior change wheel intervention design process

Intervention functions	COM-B components served by the intervention functions	Selected behavior change techniques	Policy categories through which behavior change techniques can be delivered	Mode of delivery
Modeling	Social opportunity	Demonstration of the behavior Adding objects to the environment Prompts/cues Credible source Information about health consequences Instruction on how to perform behavior Pros and cons	Communication/marketing	Leaflets
Environmental restructuring	Physical opportunity Social opportunity			
Persuasion	Reflective motivation			
Education	Psychological capability Reflective motivation			
Enablement	Psychological capability Physical opportunity Social opportunity			

**Table 2:** Overview of the intervention design

Behavior change technique	Definition	Examples of use
Pros and cons	Advise the person to identify reasons for wanting (pros) or not wanting (cons) to change behavior	A list of the benefits of bowel scope screening was added to the leaflet
Demonstration of the behavior	Provide an observable sample of the performance of the behavior, directly in person or indirectly (e.g., via film, pictures) for the person to aspire to or imitate	Testimonials of people who had performed the behavior were added to the leaflet
Credible source	Present verbal or visual communication from a credible source in favor or against the behavior	A primary care endorsement from the General Practice Cancer Lead endorsing the National Health Service Bowel Scope Screening program was added to the leaflet
Prompts/cues	Introduce or define environmental or social stimulus with the purpose of prompting or cueing the behavior. The prompt or cue would normally occur at the time or place of performance	A prompt was added to the intervention strategy by developing a “self-referral” reminder letter and a “follow-up” reminder letter
Instruction on how to perform a behavior	Advise or agree on how to perform a behavior	Instructions on how to self-refer for bowel scope screening were added to the reminder letter
Adding objects to the environment	Add objects to the environment in order to facilitate performance of the behavior	Several “objects” or facilitators were added to the reminder letters, including an “appointment-request slip” and a Freepost return envelope
Information about health consequences	Provide information (e.g., written, verbal, visual) about health consequences of performing the behavior	Information about the health consequences of bowel scope screening (e.g., reduced risk of colorectal cancer incidence and death) was added to the reminder letters

Initial versions of the intervention materials were developed by Partners in Creation: a social marketing company that specializes in the development of health behavior change interventions [20]. We provided them with a brief outlining the intervention strategy/content described in Tables 1 and 2. The drafted materials were then tested in a co-design workshop in which screening eligible adults from the London boroughs of Brent and Harrow (*n* = 4; 3 men, 1 woman; aged 55–58 years) gave feedback to inform future iterations of the materials. Revised versions were then presented to individuals who were either the eligible age or approaching the eligible age for screening (*n* = 20; 12 women, 8 men, aged 50–59 years) and feedback obtained through interviews conducted by a member of the University College London (UCL) research team. The final materials used in the trial are described under Intervention Development.

#### 24-Month reminder

The 24-month reminder was a personally addressed letter from St Mark’s Bowel Cancer Screening Centre that invited recipients to make an appointment by returning an “appointment-request-slip” or calling the Freephone number for St Mark’s Bowel Cancer Screening Centre (see online Supplementary material). The reminder also gave recipients the option to express a preference for the day and time of the appointment and the gender of the practitioner performing the test.

#### Theory-based leaflet

The theory-based leaflet was a locally tailored leaflet designed to promote bowel scope screening attendance at St Mark’s Hospital in London. The leaflet included testimonials from individuals previously screened at the center, as well as a primary care endorsement of the screening test and a list of the benefits of having the test (see online Supplementary material).

#### Follow-up reminder

The follow-up reminder was a personally addressed letter from St Mark’s Bowel Cancer Screening Centre that reiterated the opportunity to self-refer for screening up until the age of 60 (see online Supplementary material). It was included on the basis that additional reminders/prompts have been shown to have benefits over and above those of single reminders used by themselves [21]. The timing for the follow-up reminder was based on the program reminder, which is sent 4 weeks after the first contact.

#### Standard information booklet

The standard information booklet was the same 16-page booklet sent with the initial invitation as part of the national screening program (available from https://www.gov.uk/government/uploads/system/uploads/attachment_data/file/423928/bowel-scope-screening.pdf). The standard information booklet was developed by King’s Health Partners, who developed the booklet in accordance with the principles put forth by England’s National Health Service informed choice initiative [22].

### Measures

Routinely available data stored on the Bowel Cancer Screening System were used to verify self-referral and attendance 4 and 12 weeks following the distribution of the 24-month self-referral reminder letter. The Bowel Cancer Screening System was also consulted to obtain the eligibility of each person, as well as their gender (male, female), area (Brent, Harrow), and initial episode status (did not respond, did not attend). For individuals who self-referred for an appointment, the Bowel Cancer Screening System was additionally consulted to obtain the method of referral (by letter, by telephone) and whether they received a pre-appointment text message and/or telephone call (coded as “received a pre-appointment reminder: yes/no”). Lastly, for individuals who attended an appointment, the Bowel Cancer Screening System was consulted to obtain the clinical outcome and thereby the proportion of people who had one or more precancerous lesions (adenomas) detected.

An area-based socioeconomic deprivation score was generated for each person by converting their postcode into a score on the 2010 Index of Multiple Deprivation [23]. Area-level Index of Multiple Deprivation scores were then categorized into tertiles of their regional distributions to enable comparisons between the most and least deprived areas.

### Sample Size

The primary outcome was the overall uptake of screening within each group 12 weeks after the delivery of the second reminder (sent 24 months after the initial invitation). A sample size of 420 men and women per trial arm was required to detect a difference in uptake from 10.7% to 17.7% [24] in the Reminder and Standard Information Booklet and Reminder and Theory-Based Leaflet groups, respectively (α = 0.05; β = 0.2). This was increased to 460 per arm to account for dropout during reminder intervals, giving a total sample size requirement of *n* = 1,380.

### Analysis

The number and percentage of patients screened within 12 weeks of the second reminder are presented with two-sided 95% confidence intervals (CIs), constructed using exact methods based on the binomial distribution. Odds ratios (ORs), adjusted ORs (aORs), and 95% CIs comparing the uptake in each group were calculated using univariable and multivariable logistic regression to adjust for baseline characteristics. Bonferroni corrections and an adjusted significance level of 0.015 were used to account for multiple comparisons. Subgroup analyses were carried out to explore possible associations between not attending a confirmed appointment and (i) baseline characteristics, (ii) method of referral, and (iii) receipt of a pre-appointment text/telephone call. The adenoma detection rate was reported using descriptive statistics. The cumulative data were analyzed on an intention-to-treat basis using SPSS (ver.24).

### Cost Analysis

We calculated the cost per additional attendee by dividing the cost of the self-referral reminder and follow-up reminder (with the standard information booklet and theory-based leaflet separately) by the number of people who attended screening at 12 and 24 months. We also performed a sensitivity analysis by calculating the range of variation of the cost estimates within the CIs of the participation rates (calculated using exact methods based on the binomial distribution).

### Ethics

The study was approved by the North-East Tyne & Wear South Research Ethics Service (Ref: 15/NE/0043) and was registered with the International Standard Randomized Controlled Trials Number Registry for transparency (trial ID: ISRCTN44293755).

## Results

### Sample Characteristics

**Fig. 1.**
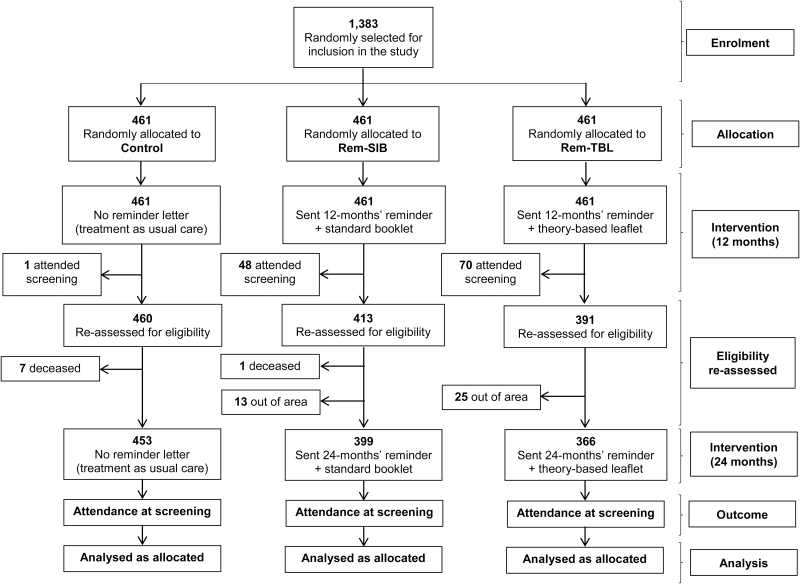
CONSORT diagram.

This study took place between February and August, 2016, with follow-up until October, 2016. In total, 1,264 (91.4%) out of 1,383 men and women from the initial sample were re-assessed for inclusion in this analysis (Fig. 1). One hundred and nineteen (8.6%) were known to have already taken part in screening and were not assessed for this reason. Of the 1,264 adults who were re-assessed, 8 (0.6%) had died, and 38 (2.8%) were no longer registered with a general practice in the London boroughs of Brent and Harrow, leaving a total sample size of 1,218 men and women who were eligible for inclusion across all three study groups (control, *n* = 453; Reminder and Standard Information Booklet, *n* = 399; Reminder and Theory-Based Leaflet, *n* = 366).

The basic attributes of each group are presented in Table 3. All participants were aged 57 because of the study design. Most (53.4%) were females (*n* = 650), registered with a general practice in the London borough of Brent (*n* = 816; 67.0%), and did not respond to the initial invitation (*n* = 1,072, 88.0%).

**Table 3:** Baseline characteristics

	Control (*n* = 453)	Rem-SIB (*n* = 399)	Rem-TBL (*n* = 366)	Total (*n* = 1,218)	^*χ*2^ (*p* Value)
Gender *n* (%)					
Female	255 (56.3)	213 (53.4)	182 (49.7)	650 (53.4)	3.51 (.173)
Male	198 (43.7)	186 (46.6)	184 (50.3)	568 (46.6)	
Area *n* (%)					
Brent	300 (66.2)	259 (64.9)	257 (70.2)	816 (67.0)	2.62 (.269)
Harrow	153 (33.8)	140 (35.1)	109 (29.8)	402 (33.0)	
Tertile of deprivation (Index of Multiple Deprivation Score) *n* (%)					
Tertile 1 (0.00–17.68)	148 (32.7)	128 (32.1)	104 (28.4)	380 (31.2)	2.14 (.710)
Tertile 2 (17.69–27.50)	164 (36.2)	141 (35.3)	142 (38.8)	447 (36.7)	
Tertile 3 (27.51–80)	137 (30.2)	126 (31.6)	115 (31.4)	378 (31.0)	
Missing	4 (0.9)	4 (1.0)	5 (1.4)	14 (1.1)	
Initial episode status *n* (%)					
Initial nonresponder	404 (89.2)	342 (85.7)	326 (89.1)	1,072 (88.0)	2.98 (.226)
Initial nonattender	49 (10.8)	57 (14.3)	40 (10.9)	146 (12.0)	

*Rem-SIB* Reminder and Standard Information Booklet; *Rem-TBL* Reminder and Theory-Based Leaflet.

### Uptake (24-Month Reminder)

In total, 50 (4.1%) men and women who received the 24-month reminder attended a screening appointment across all three study groups. A further 7 (0.6%) made an appointment, but either did not attend (*n* = 4) or cancelled (*n* = 3), leaving 1,161 (95.3%) individuals who neither made nor attended an appointment.

The percentage of people who booked and attended an appointment within each group was 0.4% (*n* = 2, 95% CI = 0.0–1.6), 4.8% (*n* = 19, 95% CI = 2.9–7.3), and 7.9% (*n* = 29, 95% CI = 5.4–11.2) in the control, Reminder and Standard Information Booklet, and Reminder and Theory-Based Leaflet groups respectively. Sending a second self-referral reminder 24 months after the initial invitation therefore further increased screening uptake and was significantly more effective than usual care.

### Uptake (12- and 24-Month Reminder Combined)

In the combined data, we found that 169 (12.2%) men and women had booked and attended an appointment across all three study groups (Table 4). A further 43 (3.1%) made an appointment, but subsequently did not attend (*n* = 25) or canceled (*n* = 18), leaving 1,171 (84.7%) who neither made nor attended an appointment. There was strong evidence of differences in booked and attended appointments between the reminder groups and the control (Table 5). A total of 67 individuals (14.5%) in the Reminder and Standard Information Booklet group and 99 individuals (21.5%) in the Reminder and Theory-Based Leaflet group attended an appointment, compared with only 3 (0.7%) in the control (OR = 25.96, 95% CI = 8.10–83.18, *p* < .001 and OR = 41.75, 95% CI = 13.13–132.76, *p* < .001 for the Reminder and Standard Information Booklet and Reminder and Theory-Based Leaflet groups, respectively). There was also strong evidence of a difference in uptake between the reminder groups, with individuals in the Reminder and Theory-Based Leaflet group being significantly more likely to attend an appointment than individuals in the Reminder and Standard Information Booklet group (OR = 1.61, 95% CI = 1.14–2.26, *p =* .006).

**Table 4:** Uptake at 12 and 24 months by trial arm

	Uptake % (95% CI)	
	12 Months	24 Months
Control (*n* = 461)	0.2 (0.0%–1.2%)	0.7 (0.2%–2.0%)
Rem-SIB (*n* = 461)	10.4 (7.8%–13.6%)	14.5 (11.4%–18.1%)
Rem-TBL (*n* = 461)	15.2 (12.1%–18.8%)	21.5 (17.8%–25.5%)

*CI* confidence interval; *Rem-SIB* Reminder and Standard Information Booklet; *Rem-TBL* Reminder and Theory-Based Leaflet.

**Table 5:** Self-referral and uptake by trial arm (12 and 24 months combined)

	*n* (%)	Unadjusted OR (95% CI)	Adjusted OR (95% CI)
Made an appointment comparisons			
Control vs. Rem-SIB	3 vs. 83 (0.7 vs. 18.0)	33.52** (10.51–106.92)	33.9** (10.60–108.36)
Control vs. Rem-TBL	3 vs. 126 (0.7 vs. 27.3)	57.42** (18.12–182.00)	65.25** (20.48–207.90)
TMR-SIB vs. Rem-TBL	83 vs. 126 (18.0 vs. 27.3)	1.71** (1.25–2.34)	1.93** (1.39–2.66)
Attended an appointment comparisons			
Control vs. Rem-SIB	3 vs. 67 (0.7 vs. 14.5)	25.96** (8.10–83.18)	26.14** (8.14–83.95)
Control vs. Rem-TBL	3 vs. 99 (0.7 vs. 21.5)	41.75** (13.13–132.76)	46.91** (14.68–149.93)
TMR-SIB vs. Rem-TBL	67 vs. 99 (14.5 vs. 21.5)	1.61* (1.14–2.26)	1.80** (1.26–2.55)

*n* = 461 for all groups reported. Adjusted ORs and 95% CIs are adjusted for gender, area, deprivation, and initial episode status.

*ORs* odds ratios; *CI* confidence interval; *Rem-SIB* Reminder and Standard Information Booklet; *Rem-TBL* Reminder and Theory-Based Leaflet.

*p ≤ .01; **p ≤ .001.

Results were similar after adjusting for baseline characteristics in the multivariable analysis (Table 5), with strong evidence of differences in uptake between the reminder groups and the control (Reminder and Standard Information Booklet vs. control: aOR = 26.14, 95% CI = 8.14–83.95, *p* < .001; Reminder and Theory-Based Leaflet vs. control: aOR = 46.91, 95% CI = 14.68–149.93, *p* < .001). After adjusting for baseline characteristics, there remained a significant difference in participation between intervention groups, with individuals in the Reminder and Theory-Based Leaflet group being more likely to book and attend an appointment than individuals in the Reminder and Standard Information Booklet group (aOR = 1.80, 95% CI = 1.26–2.55; *p* < .001). There was also strong evidence of a difference in uptake by initial episode status after adjusting for study group and other baseline characteristics, with former nonattenders (i.e., people who did not attend) being more likely to book and attend an appointment than former nonresponders (i.e., people who did not respond); uptake was 11.4% and 20.3%, respectively (aOR = 2.60, 95% CI = 1.55–4.36; *p* < .001). There was no evidence of an association between screening uptake and gender, regional Index of Multiple Deprivation tertile, or area (Table 6).

**Table 6:** Self-referral and uptake by baseline characteristics (12 and 24 months combined)

	Made an appointment *n* (%)	Unadjusted OR (95% CI)	Adjusted OR (95% CI)	Attended an appointment *n* (%)	Unadjusted OR (95% CI)	Adjusted OR (95% CI)
Gender						
Female^a^ (*n* = 727)	109 (15.0)	–	–	82 (11.3)	–	–
Male (*n* = 656)	103 (15.7)	1.06 (0.79–1.42)	0.96 (0.71–1.32)	87 (13.3)	1.20 (0.87–1.66)	1.14 (0.81–1.60)
Area						
Brent^a^ (*n* = 926)	134 (14.5)	–	–	103 (11.1)	–	–
Harrow (*n* = 457)	78 (17.1)	1.22 (0.90–1.65)	1.26 (0.84–1.89)	66 (14.4)	1.35 (0.97–1.88)	1.44 (0.93–2.24)
Deprivation						
Tertile 1^a^ (*n* = 429)	70 (16.3)	–	–	58 (13.5)	–	–
Tertile 2 (*n* = 505)	74 (14.7)	0.88 (0.62–1.26)	0.97 (0.63–1.49)	55 (10.9)	0.78 (0.53–1.16)	0.92 (0.58–1.48)
Tertile 3 (*n* = 435)	67 (15.4)	0.93 (0.65–1.35)	1.09 (0.68–1.76)	56 (12.9)	0.95 (0.64–1.40)	1.22 (0.73–2.04)
Initial episode status						
Initial nonresponder^a^ (*n* = 1,255)	181 (14.4)	–	–	143 (11.4)	–	–
Initial nonattender (*n* = 128)	31 (24.2)	1.90* (1.23–2.93)	2.67** (1.63–4.37)	26 (20.3)	1.98* (1.25–3.15)	2.60** (1.55–4.36)

Adjusted ORs and 95% CIs are adjusted for trial arm and all other covariates in the table.

*OR* odds ratio; *CI* confidence intervals; *Rem-SIB* Reminder and Standard Information Booklet; *Rem-TBL* Reminder and Theory-Based Leaflet.

^**a**^Reference category.

*p ≤ .01; **p ≤ .001.

### Confirmed Appointments (12- and 24-Month Reminder Combined)

A total of 43 individuals booked an appointment but did not attend. A significant difference in attendance among people who self-referred was observed between men and women (84.4% vs. 74.5%), with men being more likely to attend their appointment than women (aOR = 2.06, 95% CI = 1.01–4.23, *p* = .05). A similar difference in uptake was observed between people who received a pre-appointment reminder and people who did not (83.6% vs. 73.6%), although this did not reach statistical significance in the multivariable analysis (aOR = 1.70; 95% CI = 0.84–3.44, *p* = .14). There was no evidence of differences in nonattendance for any of the other variables included in the analysis, including initial episode status, method of referral and area (see online Supplementary material).

### Adenoma Detection Rate (12- and 24-Month Reminder Combined)

Of the 169 men and women who attended an appointment and were screened, 14 (8.3%) had one or more adenomas detected, 7 of whom had adenomas that met the clinical criteria for colonoscopy and subsequently underwent further examination. One person was diagnosed with cancer and was referred for treatment because of their diagnosis. In the multivariable regression (see online Supplementary material), there were no statistical differences in the proportion of individuals who had adenomas detected by trial arm or baseline characteristics (all *p* values >.05).

### Costs

The estimated cost of the interventions per additional person attending screening at 12 months were £8.37 (range: £6.38–£11.17) in the Reminder and Standard Information Booklet group and £8.75 (range: £7.05–£11.14) in the Reminder and Theory-Based Leaflet group (see online Supplementary material for a breakdown of the intervention costs for each group). Costs for both interventions were significantly higher at 24 months (95% CIs did not overlap), with at an estimated cost per additional person attending screening of £18.31 (range: £12.00–£29.00) in the Reminder and Standard Information Booklet group and £16.93 (range: £11.97–£24.55) in the Reminder and Theory-Based Leaflet group (see online Supplementary material for a breakdown of the intervention costs).

## Discussion

The results of this study provide strong evidence to support the use of a second self-referral reminder within the National Health Service bowel scope screening program and highlight an additional benefit to including a bespoke theory-based leaflet designed using the Behavior Change Wheel (the overall uptake was 0.7%, 14.5%, and 21.5% in the control, Reminder and Standard Information Booklet and Reminder and Theory-Based Leaflet groups, respectively).

At the current rate of attendance (43%) [4], the inclusion of two self-referral reminders within the National Health Service bowel scope screening program would increase uptake by ~8–12 percentage-points (estimated by multiplying the proportion of adults not attending an initial appointment [57%] by the proportion of adults attending an appointment following the delivery of the 24-month reminder with either the standard information booklet [14.5%] or the theory-based leaflet [21.5%]), depending on which of the two leaflets were adopted. Given that uptake was consistent between men and women, as well as between tertiles of area-level deprivation, it seems unlikely that implementing these reminders with either leaflet would exacerbate existing inequalities in uptake [4]. Indeed, it is possible that implementing these reminders could in fact reduce inequalities in uptake, given that the proportion of nonparticipants living in the most deprived quintile of areas is greater than the proportion living in the least deprived quintile of areas (48% vs. 68%) [4].

While uptake did not vary by gender or tertile of area-level deprivation, it did vary by initial episode status, with initial nonattenders being more likely to book and attend an appointment than initial nonresponders (20.3% vs. 11.4%). One possible explanation for this is that initial nonattenders (who perceive fewer barriers and more benefits to screening than initial nonresponders) are qualitatively similar to screened adults, but have difficulty translating their intentions into action due to circumstantial aspects, such as poor health [25]. Indeed, previous research by Ferrer and colleagues [26] has shown that participation in colorectal cancer screening is a behavioral process comprised of several qualitatively distinct stages through which individual transition based on their readiness to screen. Each stage is thought to be strongly associated with a specific set of attitudes and beliefs toward the test, and it may be that the interventions used in our study were more effective at facilitating forward stage transitions in initial nonattenders by addressing issues that were specific to them.

Our study also found that, among individuals who made an appointment, women were less likely to attend screening than men (74.5% vs. 84.5%). This was consistent with previous research in which women who stated that they “probably would” or “definitely would” attend screening were less likely to attend than their male counterparts [25]. Given its position within the screening pathway, it seems likely that these differences in uptake between men and women are due to the enema, which has previously been reported as a major barrier for women, but not men [27].

In terms of the clinical findings, the adenoma detection rate (8.3%) was similar to that of initial attenders (i.e., 9.8%) [7]. The rate was also consistent across reminder groups, irrespective of the information used, suggesting that both materials were effective at attracting individuals with colorectal pathology. With regards to reminder intervals (i.e., 12 months vs. 24 months), the study was underpowered to detect whether the total number of adenomas detected increased. Further studies with larger sample sizes are required to test this.

Finally, few previous studies have been able to demonstrate the added value of theory-based materials on colorectal cancer screening rates [28], particularly with regards to flexible sigmoidoscopy screening [8]. The finding that the theory-based leaflet (albeit predominantly with the first reminder) used in this study was effective is, therefore, highly encouraging. Not only does it demonstrate that such materials designed using theory can be effective, but that they can be implemented in ways that do not contravene General Medical Council guidelines for informed consent (e.g., by being sent after the full suite of information has been received by the patient). Furthermore, the findings from the present study provide evidence to support the use of the Behavior Change Wheel as a framework for developing theory-based interventions. Had we used another approach, the study materials may have been similarly ineffective to those described in the previous literature.

### Strengths

This study had several strengths. First, it used a randomized design, which is considered the gold standard in terms of evaluating the effectiveness of public health interventions [29]. Second, it is the first study to examine whether self-referral reminders can increase the uptake of bowel scope screening and, as such, is the first study to show that these are effective without being vulnerable to bias and confounding present in other studies. Finally, the study setting (St Mark’s Bowel Cancer Screening Centre) serves an ethnically diverse population from a range of socioeconomic areas and, as a result, the findings are likely to be generalizable to other London boroughs and international urban settings struggling to reach the European target for acceptable participation [30].

### Limitations

As well as several strengths, this study had a number of important limitations: the main one being that we only examined the impact of the interventions at a single center and another being that we only selected a proportion of former nonparticipants for inclusion in the trial—not the entire eligible population. An important next step, therefore, would be to investigate the feasibility of rolling out these reminders across the entire eligible cohort of nonparticipants. On the basis that the first reminder was effective, the English National Health Service have commissioned St Mark’s Hospital to carry out this work at the London center. It is our hope that after the publication of the current findings, the English National Health Service will also commission St Mark’s Hospital to implement and evaluate the use of a 24-month reminder as well.

Another important caveat of our study is that, while our leaflet was largely driven by theory-based insights, some of its characteristics were based on anecdotal evidence, or previous empirical observations. For example, the theory-based leaflet was shorter and had a lower readability score on the basis of previous research highlighting barriers to engaging with written information about colorectal cancer screening by individuals with both low and high literacy [31, 32]. Without additional studies exploring the reasons why people self-referred for screening (in both groups), it is not possible to say why the theory-based leaflet was more effective. Future studies using questionnaires to examine which of the COM-B components were affected by the study materials could also help elucidate how the interventions facilitated behavior change. A factorial randomized controlled trial comparing multiple versions of the theory-based leaflet would ultimately be needed to disentangle which of the behavior change techniques helped to facilitate behavior change and thereby self-referral and uptake.

Finally, our study was limited to routine data stored on the Bowel Cancer Screening System. As such, it was not possible to include other potential predictors of responding to the screening invite and attendance at screening (e.g., previous bowel symptoms, and ethnicity) [25].

## Conclusion

Sending former nonparticipants a self-referral reminder 12 and 24 months after their initial invitation was effective at improving uptake and was enhanced by the inclusion of a theory-based leaflet developed using the Behavior Change Wheel. Future studies should focus on the feasibility of implementing these interventions across multiple centers and the wider population of eligible adults.

## Supplementary Material

Supplementary material is available at *Annals of Behavioral Medicine* online.

## Supplementary Material

Supplementary Materials
