# Supplementary material for: Use of Two Self-referral Reminders and a Theory-Based Leaflet to Increase the Uptake of Flexible Sigmoidoscopy in the English Bowel Scope Screening Program: Results From a Randomized Controlled Trial in London
Source: Ann Behav Med. 2018 Jan 15;52(11):941–51. doi: 10.1093/abm/kax068 (PMC6196365; doi:10.1093/abm/kax068)
Supplement: Supplementary Materials [file kax068_suppl_supplementary_materials.docx]

| **Table S1.** *Worksheet 1 - Define the problem in behavioural terms.* | |
| --- | --- |
| Question | Answer |
| What behaviour? | Uptake of bowel scope screening. |
| Where? | St Mark’s Hospital. |
| Who? | Eligible men and women aged 55-59. |

| **Table S2.** *Worksheet 2 - Select target behaviour.* | | | | |
| --- | --- | --- | --- | --- |
| **Task 1.** Generate a list of candidate target behaviours that could bring about the desired outcome | | | | |
| **Intervention aim:** Improve uptake of bowel scope screening among eligible adults at St Mark’s Hospital. | | | | |
| **Candidate target behaviours:**   - 1. Confirmation of initial appointment offered   2. Attendance of confirmed appointment   3. Self-referral | | | | |
| **Task 2.** Prioritise candidate target behaviours | | | | |
| Potential target behaviours relevant to improving uptake of bowel scope screening at St Mark’s Hospital | How much of an impact will changing the behaviour have on desired outcome? | How likely it is that behaviour can be changed? | How likely is it that the behaviour will have an impact on other related behaviours? | How easy will it be to measure the behaviour? |
| 1) Confirmation of initial appointment | A big impact, 80% of people who confirm their initial appointment attend | Not very likely, can’t change invitation materials | Not likely to change attendance of confirmed appointments or self-referral | Very easy – data available on the Bowel Cancer Screening System |
| 2) Attendance of confirmed appointment | A moderate impact, if everyone who confirmed their appointment attended uptake would increase by 10% | Not very likely, can’t change existing reminder materials | Not likely to change self-referral or acceptance of initial appointment offered | Very easy – data available on the Bowel Cancer Screening System |
| 3) Self-referral | A big impact, half of non-participants interviewed in a recent study indicated they would consider screening in the future | Very likely, can introduce non-participant interventions | Not likely to change acceptance of initial appointment offered or attendance of confirmed appointments | Very easy – data available on the Bowel Cancer Screening System |
| **Selected target behaviour:** Self-referral | | | | |

| **Table S3.** *Worksheet 3 – Specify the target behaviour.* | |
| --- | --- |
| **Task 1.** Describe the target behaviour according to who needs to do what, when, where, how often and with whom | |
| Question | Answer |
| What is the target behaviour? | Self-referral |
| Who needs to perform the behaviour? | Bowel scope screening non-participants |
| What do they need to do to achieve the desired change? | Book an appointment |
| Where do they need to do it? | They can do it from anywhere |
| How often do they need to do it? | Once, before the age of 60 |
| With whom do they need to do it? | A member of staff at St Mark’s Hospital |

| **Table S4.** *Worksheet 4 – Identify what needs to change.* | | | |
| --- | --- | --- | --- |
| **Task 1.** Describe the target behaviour according to who needs to do what, when, where, how often and with whom | | | |
| COM-B components | Transtheoretical domains framework domains linking to COM-B components | What needs to happen for the target behaviour to occur? | Is there a need for change? |
| Physical capability | Physical skills | The appointment needs to be convenient | No, people who self-refer for screening are able to choose the day and time of their appointment |
| Psychological capability | Knowledge | The person needs to know the harms and benefits of screening | Yes, 63% of colorectal cancer screening non-participants report that they did not read the information leaflet (Koboyashi et al., 2016), and 86% of all age-appropriate adults do not know that bowel scope screening helps prevent bowel cancer (Chorley et al., 2017) |
|  | Cognitive and interpersonal skills | The person needs to be able to read and understand the information provided to them | Yes, 63% of non-participants do not read the information currently used by the programme (Kobayashi et al., 2016) |
|  | Memory attention and decision processes | Not relevant | - |
|  | Behavioural regulation | Not relevant | - |
| Physical opportunity | Environmental context and resources | Non-participants need to be reminded of the procedures for self-referral | Yes, there are currently no cues reminding non-participants of the procedures for self-referral |
| Social opportunity | Social influences | The behavioural needs to be normalised | Yes, bowel scope screening is still very novel, and therefore not yet normalised |
| Reflective motivation | Social professional role and identity | Person needs to perceive themselves as someone who looks after their health | Yes, person needs to be made aware that they are at risk of developing bowel cancer |
|  | Beliefs about capabilities | Not relevant | Not relevant |
|  | Optimism | Not relevant | Not relevant |

|  | Intentions | Person has to intend to go for bowel scope screening | Yes, person needs to translate their intentions into actions by self-referring for bowel scope screening |
| --- | --- | --- | --- |
|  | Goals | Not relevant | Not relevant |
|  | Beliefs about consequences | Person needs to believe in the effectiveness of screening | Yes, 86% of all age-appropriate adults do not know that bowel scope screening helps prevent bowel cancer (Chorley et al., 2017) |
| Automatic motivation | Reinforcement | Not relevant | Not relevant |
|  | Emotion | Not relevant | Not relevant |
| Behavioural diagnosis of the relevant COM-B components: | Psychological capability, physical opportunity, social opportunity and reflective motivation need to change in order for the behaviour to occur. | | |

| **Table S5.** *Worksheet 5 – Identify intervention functions.* | |
| --- | --- |
| Candidate intervention functions | Does the intervention function meet the APEASE criteria (affordability, practicability, effectiveness/cost-effectiveness, acceptability, side-effects/safety, equity) in the context of self-referring for an appointment after the initial invitation? |
| Modelling | Yes |
| Environmental restructuring | Yes |
| Persuasion | Yes |
| Incentivisation | No, unethical/not acceptable |
| Coercion | No, unethical/not acceptable |
| Education | Yes |
| Training | Not relevant in context of self-referral |
| Enablement | Yes |
| Restriction | Not relevant in context of self-referral |
| Selected Intervention functions: | Modelling, environmental restructuring, persuasion, education and enablement |

| **Table S6.** *Worksheet 6 – Identify policy categories.* | | | | |
| --- | --- | --- | --- | --- |
| Intervention function | COM-B component | Potentially useful policy categories | Does the policy category meet the APEASE criteria in the context of self-referring for an appointment after the initial invitation? |  |
| Modelling | Social opportunity  Automatic motivation | Communication/marketing | Yes |  |
|  |  | Service provision | Not relevant in self-referral context |  |
| Environmental restructuring | Physical opportunity  Social opportunity  Automatic motivation | Guidelines | Possible in the long term, but not present |  |
|  |  | Fiscal measures | No, not acceptable. |  |
|  |  | Regulation | Not relevant in the self-referral context |  |
|  |  | Legislation | Not relevant in the self-referral context |  |
|  |  | Environmental/social planning | Not relevant in the self-referral context |  |
| Persuasion | Automatic motivation  Reflective motivation | Communication/marketing | As above |  |
|  |  | Guidelines | As above |  |
|  |  | Regulation | As above |  |
|  |  | Legislation | As above | |
|  |  | Service provision | As above | |
| Education | Psychological capability  Reflective motivation | Communication/marketing | As above | |
|  |  | Guidelines | As above | |
|  |  | Regulation | As above | |
|  |  | Legislation | As above | |
|  |  | Service provision | As above | |
| Enablement | Physical capability  Psychological capability  Physical opportunity  Social opportunity  Automatic motivation | Guidelines | As above | |
|  |  | Fiscal measures | As above | |
|  |  | Regulation | As above | |
|  |  | Legislation | As above | |
|  |  | Environmental/social planning | As above | |
|  |  | Service provision | As above | |
| **Policy category selected:** Communication/marketing | | | | |

| **Table S7.** *Worksheet 7 – Identify behaviour change techniques.* | | | | |
| --- | --- | --- | --- | --- |
| Intervention function | COM-B component | Most frequently used behaviour change techniques | | Does the behaviour change technique meet the APEASE criteria in the context of self-referring for an appointment after the initial invitation? |
| Modelling | Social opportunity  Automatic motivation | Demonstration of the behaviour | | Yes |
| Environmental restructuring | Physical opportunity  Social opportunity  Automatic motivation | Adding objects to the environment | | Yes |
|  |  | Prompts/cues | | Yes |
|  |  | Restructuring the physical environment | | Not relevant in the self-referral context |
| Persuasion | Automatic motivation  Reflective motivation | Credible source | | Yes |
|  |  | Information about social and environmental consequences | | Not relevant in self-referral context |
|  |  | Information about health consequences | | Yes |
|  |  | Feedback on behaviour | | No, not practicable |
|  |  | Feedback on outcome(s) of the behaviour | | Yes |
| Education. | Psychological capability  Reflective motivation | | Information about social and environmental consequences | As above |
|  |  |  | Information about health consequences | As above |
|  |  |  | Feedback on behaviour | As above |
|  |  |  | Feedback on outcomes of the behaviour | As above |
|  |  |  | Prompts/cues | As above |
|  |  |  | Self-monitoring of behaviour | Not applicable |
| Enablement | Physical capability  Psychological capability  Physical opportunity  Social opportunity  Automatic motivation | |  |  |
| **Behaviour change techniques selected:** Demonstration of the behaviour, adding objects to the environment, prompts/cues, credible source, information about health consequences and feedback on outcome(s) of the behaviour | | | | |

| **Table S8.** *Worksheet 8 – Identify mode of delivery.* | | | | |
| --- | --- | --- | --- | --- |
| Mode of delivery | | | | Does the mode of delivery meet the APEASE criteria in the context of self-referring for an appointment after the initial invitation? |
| Face-to-face | Individual | | | No, not likely to be affordable, practicable or cost-effective |
|  | Group | | | No, not likely to be affordable, practicable or cost-effective |
| Distance | Population-level | Broadcast media | TV | No, not likely to be affordable or practicable |
|  |  |  | Radio | No, not likely to be affordable or practicable |
|  |  | Outdoor media | Billboard | No, not likely to be practicable or equitable |
|  |  |  | Poster | No, not likely to be equitable |
|  |  | Print media | Newspaper | No, not likely to be equitable |
|  |  |  | Leaflets | Yes |
|  |  | Digital media | Internet | No, not likely to be equitable |
|  |  |  | Mobile phone app | No, not likely to be equitable |
|  | Individual-level | Phone | Phone call | No, not likely to be cost-effective |
|  |  |  | Mobile phone text | No, not likely to be equitable or practicable (screening centre does not have access to telephone numbers) |

**Figure S2.**

*24 Month self-referral reminder letter.*

**
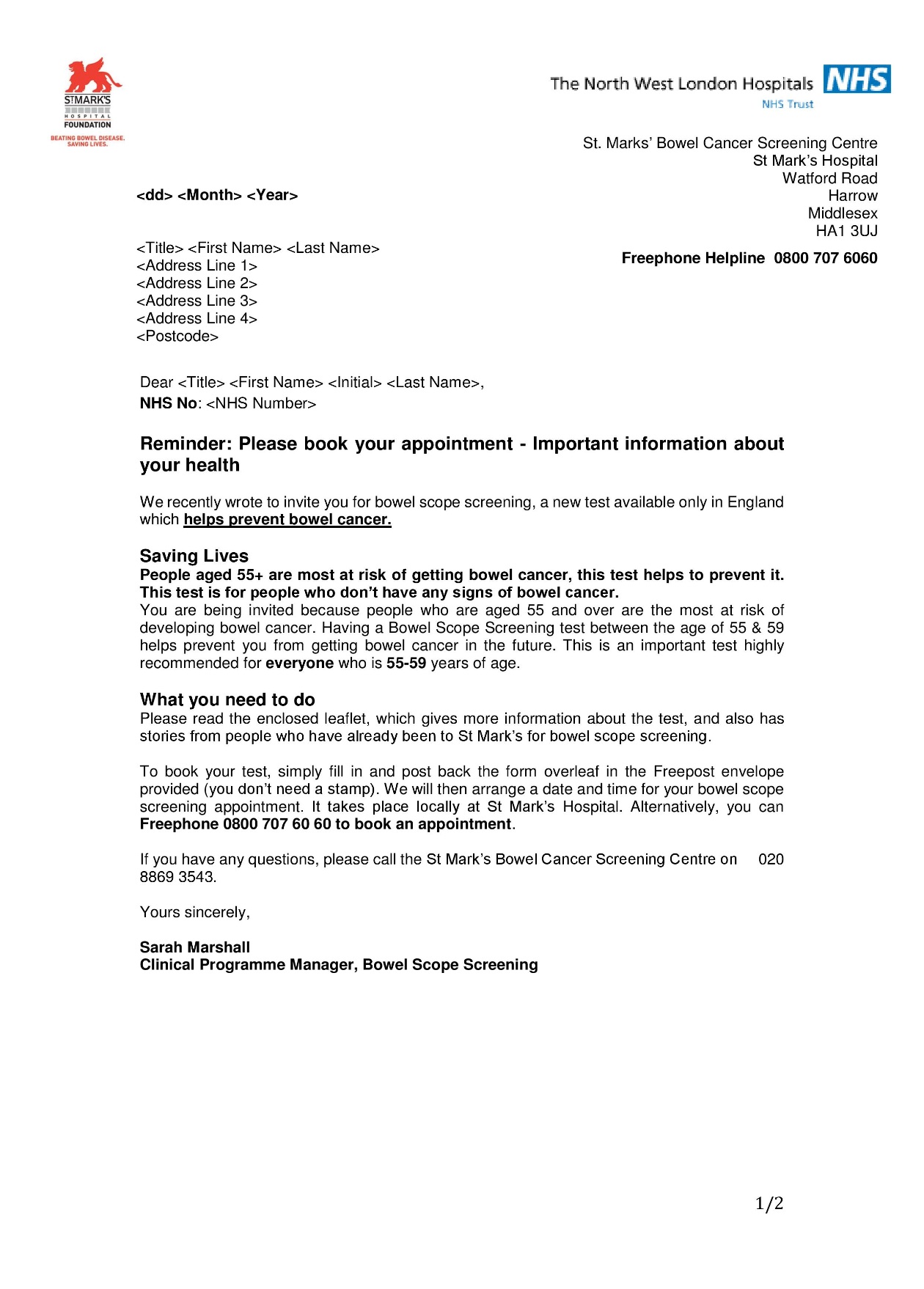
**

**
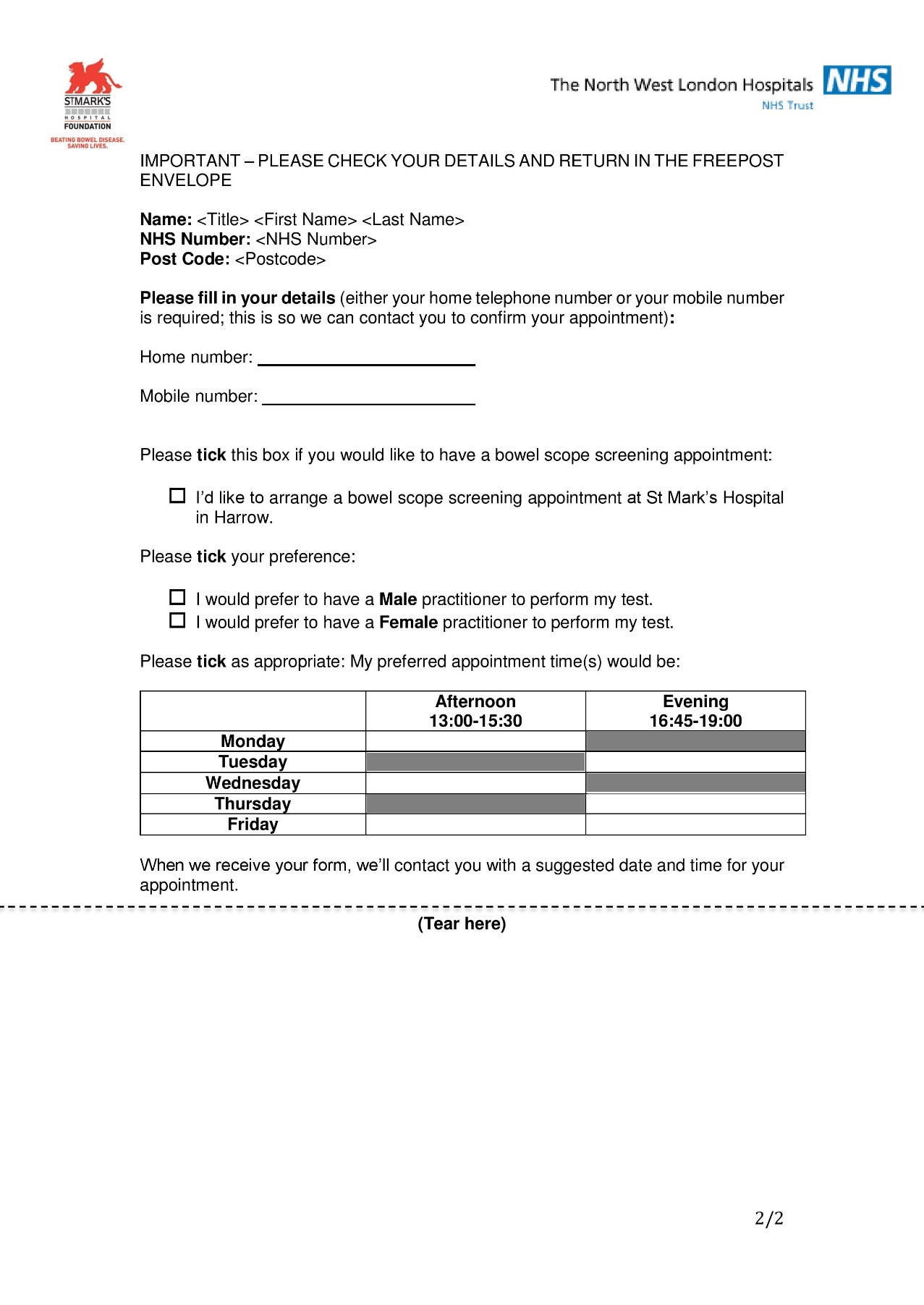
**

**Figure S3.**

*Theory-based Leaflet.*

**
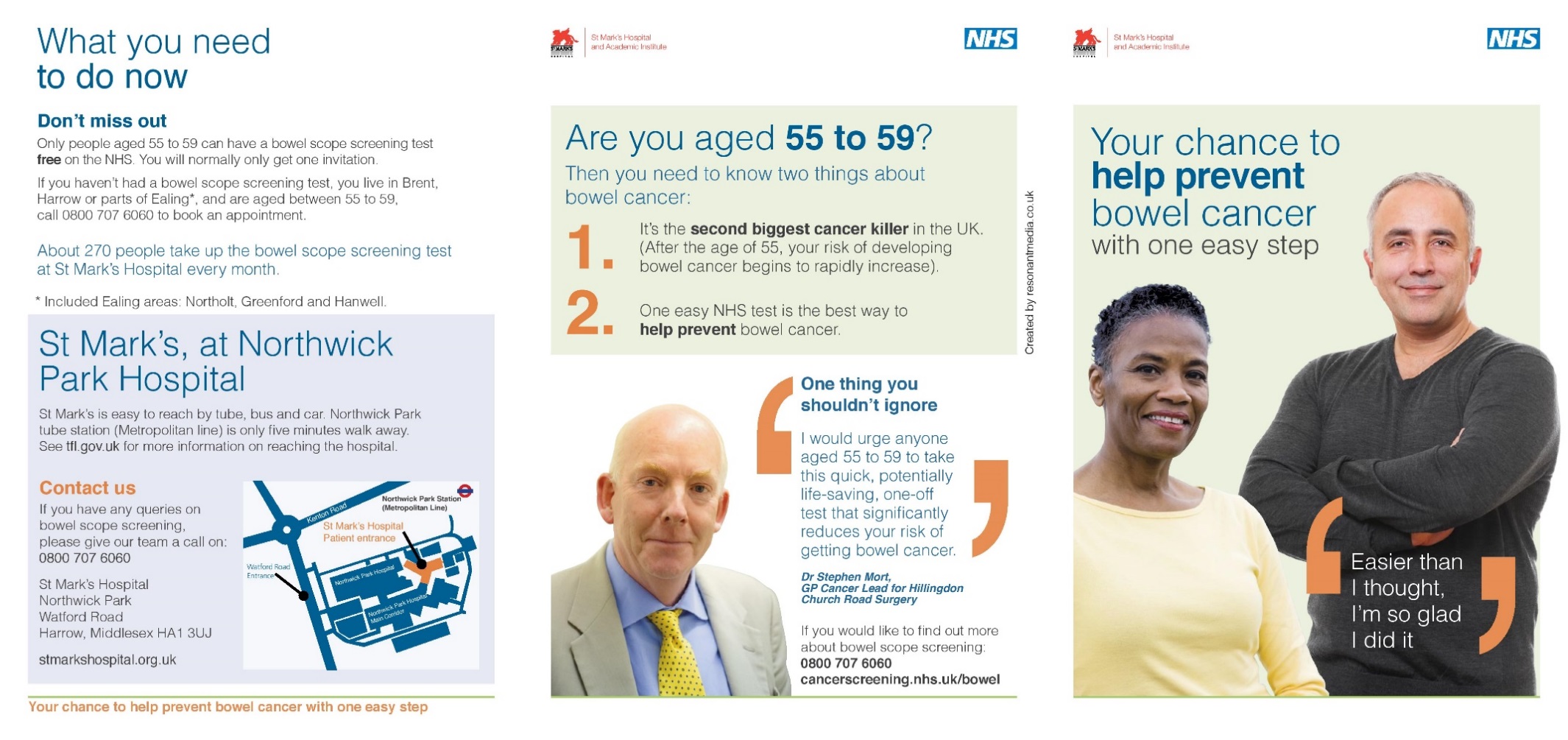
**

**
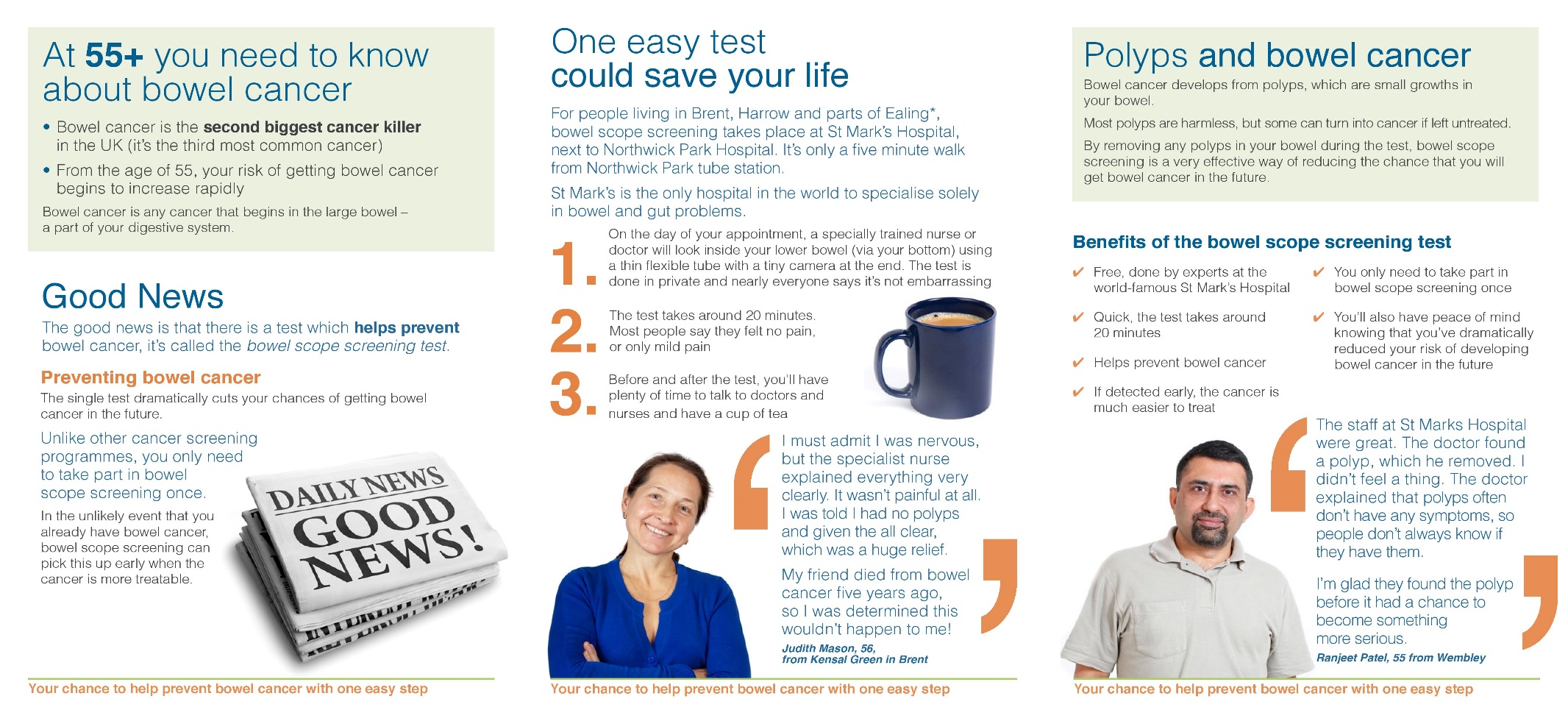
**

**Figure S4.**

*Follow-up self-referral reminder letter.*

**
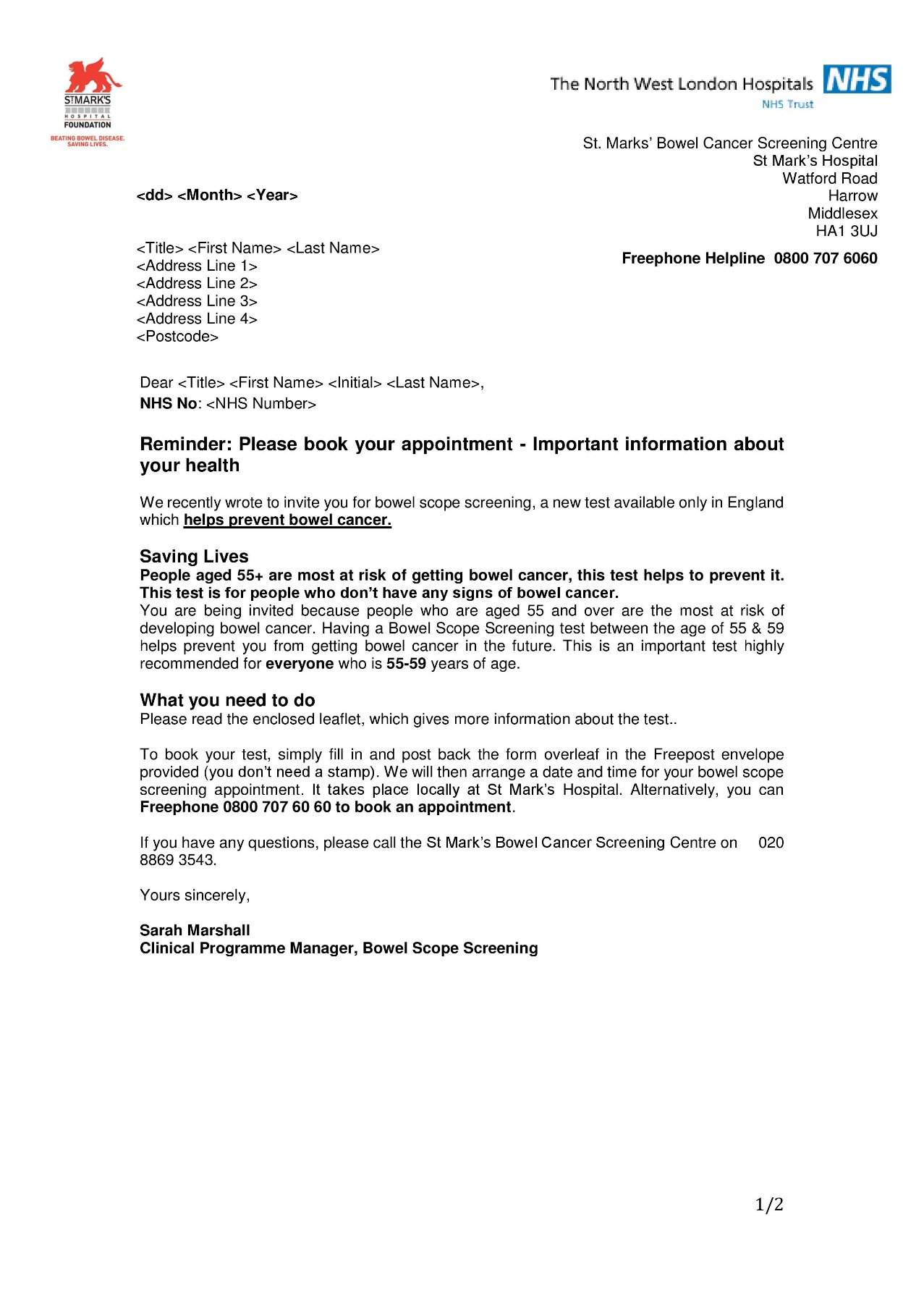
**

**
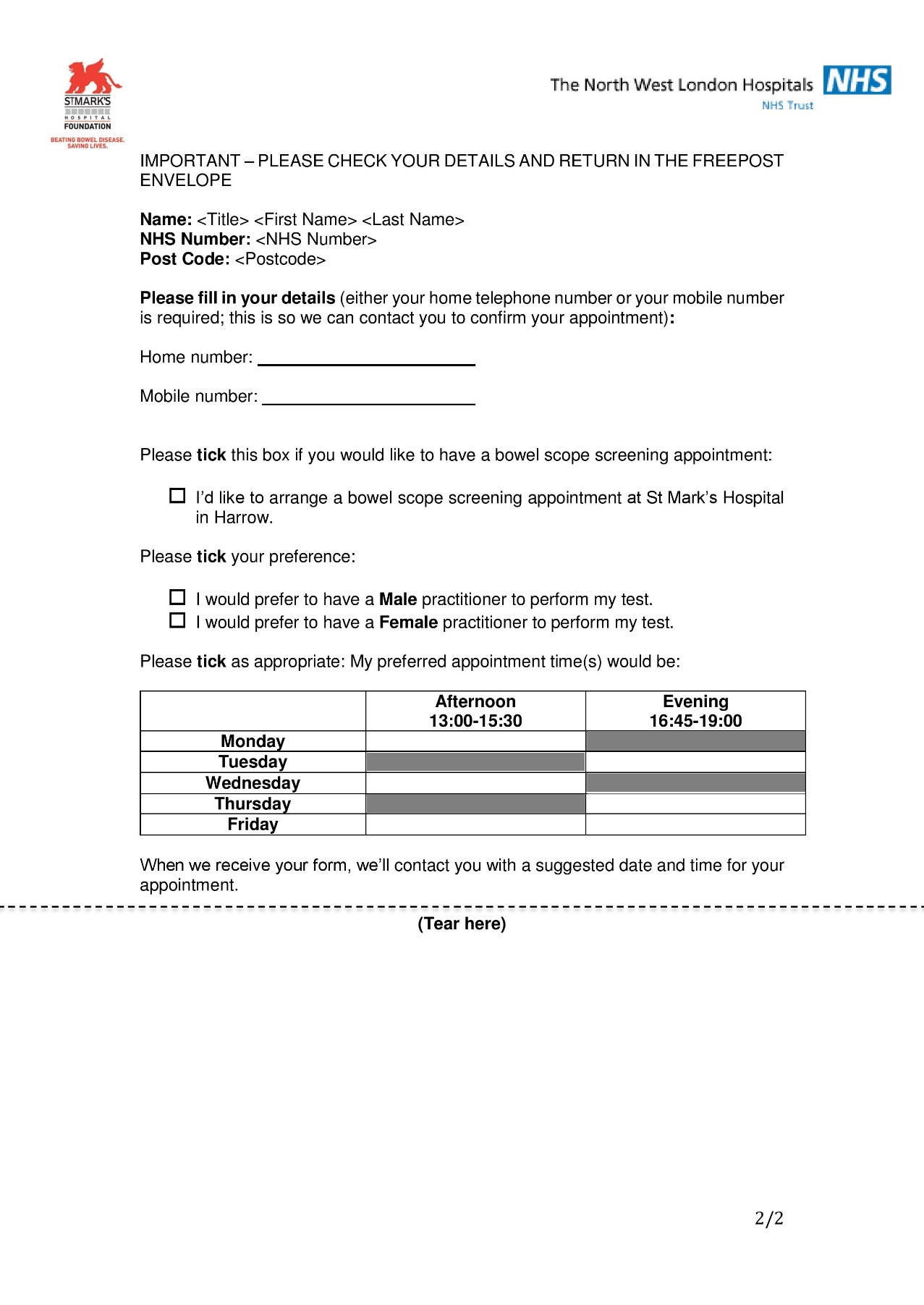
**

| **Table S9.** *Uptake of self-referred appointment by baseline characteristics and trial arm (univariate and multivariate regression)* | | | |
| --- | --- | --- | --- |
|  | Attended an appointment  *n (%)* | Unadjusted ORs  (95%CIs) | Adjusted ORs  (95%CIs) |
| Treatment group comparisons | | | |
| Reminder and Standard Information Booklet^a^  (n=83) | 67  (80.7) | - | - |
| Reminder and Theory-Based Leaflet  (n=126) | 99  (78.6) | 0.88  (0.44-1.75) | 0.85  (0.41-1.77) |
| Gender | | | |
| Women^a^  (*n*=106) | 79  (74.5%) | - | - |
| Men  (*n*=103) | 87  (84.5) | 1.86  (0.93-3.70) | 2.06*  (1.01-4.23) |
| Area | | | |
| Brent^a^  (*n*=132) | 101  (76.5) | - | - |
| Harrow  (*n*=77) | 65  (84.4) | 1.66  (0.80-3.47) | 1.74  (0.68-4.40) |
| Deprivation | | | |
| Tertile 1^a^  (*n*=69) | 57  (82.6) | - | - |
| Tertile 2  (*n*=74) | 55  (74.3) | 0.61  (0.27-1.37) | 0.83  (0.32-2.17) |
| Tertile 3  (*n*=65) | 54  (83.1) | 1.03  (0.42-2.54) | 1.56  (0.51-4.74) |

| Initial episode status | | | |
| --- | --- | --- | --- |
| Non-responder^a^  (*n*=180) | 142  (78.9) | - | - |
| Non-attender  (*n*=29) | 24  (82.8) | 1.29  (0.46-3.59) | 1.08  (0.36-3.18) |
| Referral method | | | |
| Returned slip  (*n*=175) | 138  (78.9%) | - | - |
| Telephoned  (*n*=34) | 28  (82.4) | 1.25  (0.48-3.25) | 1.70  (0.84-3.44) |
| Received a pre-appointment reminder by text and/ or by phone | | | |
| No  (*n*=87) | 64  (73.6) | - | - |
| Yes  (*n*=122) | 102  (83.6) | 1.83  (0.93-3.60) | 0.79  (0.29-2.18) |
| **^a^**Reference category  Table excludes data from the control arm which included three self-referred appointments only  Abbreviations: OR = odds ratio; CI = confidence intervals  **P*<0.05 | | | |

| **Table S10.** *Adenomas detected by trial arm and baseline characteristics - 12 & 24 months data combined (univariate and multivariate regression).* | | | |
| --- | --- | --- | --- |
|  | Adenomas detected  n (%) | Unadjusted OR  (95% CI) | Adjusted OR^1^  (95% CI) |
| Group | | | |
| Reminder and Standard Information Booklet^a^  (n = 65) | 3  (4.6) | - | - |
| Reminder and Theory-Based Leaflet  (n = 93) | 11  (11.8) | 2.77  (0.74 - 10.36) | 2.75  (0.70 - 10.78) |
| Gender | | | |
| Women^a^  (n = 76) | 7  (9.2) | - | - |
| Men  (n = 82) | 7  (8.5) | 0.92  (0.31 - 2.76) | 0.87  (0.27 - 2.76) |
| CCG | | | |
| Brent^a^  (n = 96) | 10  (10.4) | - | - |
| Harrow  (n = 62) | 4  (6.5) | 0.59  (0.18 - 1.98) | 0.82  (0.19 - 3.53) |
| Deprivation | | | |
| Tertile 1^a^  (n = 53) | 2  (3.8) | - | - |
| Tertile 2  (n = 54) | 8  (14.8) | 4.44  (0.90 - 21.96) | 4.48  (0.74 - 25.98) |
| Tertile 3  (n = 51) | 4  (7.8) | 2.17  (0.38 - 12.40) | 2.29  (0.31 - 16.95) |
| Initial episode status | | | |
| Non-responder^a^  (n = 136) | 11  (8.1) | - | - |
| Non-attender  (n = 22) | 3  (13.6) | 1.79  (0.46 - 7.02) | 2.67  (0.60 - 11.79) |
| Abbreviations: OR, Odds Ratio; CI, Confidence Interval; CCG, Clinical Commissioning Group  ^1^Adjusted ORs and 95% CIs are adjusted for all other co-variates in the table  **^a^**Reference category | | | |

| **Table S11.** *Associated costs of the reminder and standard information booklet.* | | | | | | | | |
| --- | --- | --- | --- | --- | --- | --- | --- | --- |
| Cost analysis | | | | | | | | |
|  |  | | | | 12 months’ reminder | | Follow-up reminder | |
| Item | Quantity Ordered | Cost  (per order) | Cost  (per unit) | Units  (per person) | Units  (totals) | Cost  (totals) | Units  (totals) | Cost  (totals) |
| Headed Paper | 15000 | £440.26 | £0.03 | (2) | 922 | £27.66 | 848 | £25.44 |
| A5 Envelopes | 500 | £10.08 | £0.02 | (1) | 461 | £9.22 | 424 | £8.48 |
| Pre-paid envelopes | 3000 | £224.33 | £0.075 | (1) | 461 | £34.58 | 424 | £31.80 |
| Business Reply Plus | - | - | £0.27 | N/A | 37 | £9.99 | 27 | £7.29 |
| Toner Cartridge | 1 | £194.20 | £0.00492 | (2) | 922 | £4.54 | 848 | £4.17 |
| Postage (2^nd^ Class) | - | - | £0.27 | (1) | 461 | £124.47 | 424 | £114.48 |
| Standard information booklet | - | - | £0.00 | (1) | 461 | £0.00 | 424 | £0.00 |
| Box of staples | 5,000 | £0.36 | £0.000072 | (1) | 461 | £0.03 | 424 | £0.03 |
| Total direct costs of each reminder | - | - | - | - | - | **£210.49** | - | **£191.69** |
| Total direct costs of both reminders |  |  |  |  | **£402.18** | | | |
| Cost per person sent an intervention |  |  |  |  | **£0.87**  (£402.18 / 461) | | | |
| Cost per additional screening attendee |  |  |  |  | **£8.38**  (£402.18 / 48) | | | |
| Sensitivity analysis | | | | | | | | |
| Cost per additional screening attendee  (Lower 95% CI) |  |  |  |  | **£11.17**  (£402.18 / 36) | | | |
| Cost per additional screening attendee  (Upper 95% CI) |  |  |  |  | **£6.38**  (£402.18 / 63) | | | |
| Abbreviations: CI = Confidence Interval | | | | | | | | |

| **Table S12.** *Associated costs of the reminder and theory based leaflet.* | | | | | | | | |
| --- | --- | --- | --- | --- | --- | --- | --- | --- |
| Direct costs | | | | | | | | |
|  |  | | | | 12 months’ reminder | | Follow-up reminder | |
| Item | Quantity Ordered | Cost  (per order) | Cost  (per unit) | Units  (per person) | Units  (totals) | Cost  (totals) | Units  (totals) | Cost  (totals) |
| Headed Paper | 15000 | £440.26 | £0.03 | (2) | 922 | £27.66 | 826 | £24.78 |
| A5 Envelopes | 500 | £10.08 | £0.02 | (1) | 461 | £9.22 | 413 | £8.26 |
| Pre-paid envelopes | 3000 | £224.33 | £0.075 | (1) | 461 | £34.58 | 413 | £30.98 |
| Business Reply Plus | - | - | £0.27 | N/A | 48 | £12.96 | 47 | £12.70 |
| Toner Cartridge | 1 | £194.20 | £0.00492 | (2) | 922 | £4.54 | 826 | £4.06 |
| Postage (2^nd^ Class) |  |  | £0.27 | (1) | 461 | £124.47 | 413 | £111.51 |
| Theory-based leaflet | 3000 | £711.36 | £0.237 | (1) | 461 | £109.26 | 413 | £97.88 |
| Box of staples | 5,000 | £0.36 | £0.000072 | (1) | 461 | £0.03 | 413 | £0.03 |
| Total direct costs of each reminder | - | - | - | - | - | **£322.72** | - | **£290.20** |
| Total direct costs of both reminders |  |  |  |  | **£612.92** | | | |
| Cost per person sent an intervention |  |  |  |  | **£1.33**  (£612.92 / 461) | | | |
| Cost per additional screening attendee |  |  |  |  | **£8.75**  (£612.92 / 70) | | | |
| Sensitivity analysis | | | | | | | | |
| Cost per additional screening attendee  (Lower 95% CI) |  |  |  |  | **£11.14**  (£612.92 / 55) | | | |
| Cost per additional screening attendee  (Upper 95% CI) |  |  |  |  | **£7.05**  (£612.92 / 87) | | | |
| Abbreviations: CI = Confidence Interval | | | | | | | | |

| **Table S13.** *Associated costs of the 24 months’ reminder intervention and standard information booklet.* | | | | | | | | |
| --- | --- | --- | --- | --- | --- | --- | --- | --- |
| Direct costs | | | | | | | | |
|  |  |  |  |  | 24 months reminder | | Follow-up reminder | |
| Item | Quantity Ordered | Cost  (per order) | Cost  (per unit) | Units  (per person) | Units  (totals) | Cost  (totals) | Units  (totals) | Cost  (totals) |
| Headed Paper | 15000 | £440.26 | £0.03 | (2) | 798 | £23.94 | 776 | £23.28 |
| A5 Envelopes | 500 | £10.08 | £0.02 | (1) | 399 | £7.98 | 388 | £7.76 |
| Pre-paid envelopes | 3000 | £224.33 | £0.075 | (1) | 399 | £29..93 | 388 | £29.10 |
| Business Reply Plus | - | - | £0.27 | N/A | 11 | £2.97 | 10 | £2.70 |
| Toner Cartridge | 1 | £194.20 | £0.00492 | (2) | 798 | £3.93 | 776 | £3.82 |
| Postage (2^nd^ Class) | - | - | £0.27 | (1) | 399 | £107.73 | 388 | £104.76 |
| Standard information booklet | - | - | £0.00 | (1) | 399 | £0.00 | 388 | £0.00 |
| Box of staples | 5,000 | £0.36 | £0.000072 | (1) | 399 | £0.03 | 388 | £0.03 |
| Total direct costs of each reminder | - | - | - | - | - | **£176.51** | - | **£171.45** |
| Total direct costs of both reminders |  |  |  |  | **£347.96** | | | |
| Costs per person sent an intervention |  |  |  |  | **£0.87**  (£347.96 / 399) | | | |
| Cost per additional screening attendee |  |  |  |  | **£18.31**  (£347.96 / 19) | | | |
| Sensitivity analysis | | | | | | | | |
| Cost per additional screening attendee  (Lower 95% CI) |  |  |  |  | **£29.00**  (£347.96 / 12) | | | |
| Cost per additional screening attendee  (Upper 95% CI) |  |  |  |  | **£12.00**  (£347.96 / 29) | | | |
| Abbreviations: CI = Confidence Interval | | | | | | | | |

| **Table S14.** *Associated costs of the 24 month’s reminder intervention and theory-based leaflet.* | | | | | | | | |
| --- | --- | --- | --- | --- | --- | --- | --- | --- |
| Direct costs | | | | | | | | |
|  |  |  |  |  | 24 months reminder | | Follow-up reminder | |
| Item | Quantity Ordered | Cost  (per order) | Cost  (per unit) | Units  (per person) | Units  (totals) | Cost  (totals) | Units  (totals) | Cost  (totals) |
| Headed Paper | 15000 | £440.26 | £0.03 | (2) | 732 | £21.96 | 702 | £21.06 |
| A5 Envelopes | 500 | £10.08 | £0.02 | (1) | 366 | £7.32 | 351 | £7.02 |
| Pre-paid envelopes | 3000 | £224.33 | £0.075 | (1) | 366 | £27.45 | 351 | £26.33 |
| Business Reply Plus | - | - | £0.27 | N/A | 15 | £4.05 | 19 | £5.13 |
| Toner Cartridge | 1 | £194.20 | £0.00492 | (2) | 732 | £3.60 | 702 | £3.45 |
| Postage (2^nd^ Class) |  |  | £0.27 | (1) | 366 | £98.82 | 351 | £94.77 |
| Theory-based leaflet | 3000 | £711.36 | £0.237 | (1) | 366 | £86.74 | 351 | £83.19 |
| Box of staples | 5,000 | £0.36 | £0.000072 | (1) | 366 | £0.03 | 351 | £0.03 |
| Total direct costs of each reminder | - | - | - | - | - | **£249.97** | - | **£240.98** |
| Total direct costs of both reminders |  |  |  |  | **£490.95** | | | |
| Costs per person sent an intervention |  |  |  |  | **£1.34**  (£490.95 / 366) | | | |
| Cost per additional screening attendee |  |  |  |  | **£16.93**  (£490.95 / 29) | | | |
| **Sensitivity analysis** | | | | | | | | |
| Cost per additional screening attendee  (Lower 95% CI) |  |  |  |  | **£24.55**  (£490.95 / 20) | | | |
| Cost per additional screening attendee  (Upper 95% CI) |  |  |  |  | **£11.97**  (£490.95 / 41) | | | |
| Abbreviations: CI = Confidence Interval | | | | | | | | |
